# An urban-to-rural continuum of malaria risk: new analytic approaches characterize patterns in Malawi

**DOI:** 10.1186/s12936-021-03950-5

**Published:** 2021-10-24

**Authors:** Peter S. Larson, Joseph N. S. Eisenberg, Veronica J. Berrocal, Don P. Mathanga, Mark L. Wilson

**Affiliations:** 1 Department of Epidemiology, School of Public Health, University of Michigan, 1415 Washington Heights, Ann Arbor, MI 48109 USA; 2 Department of Statistics, School of Information and Computer Sciences, University of California, Irvine, CA 92697 USA; 3 Malaria Alert Centre, College of Medicine, University of Malawi, Blantyre, Malawi; 4 Department of Community Health, College of Medicine, University of Malawi, Blantyre, Malawi

**Keywords:** Urbanicity, Environmental risk, Malaria prevention, Remote sensing, Spatial analysis

## Abstract

**Background:**

The urban–rural designation has been an important risk factor in infectious disease epidemiology. Many studies rely on a politically determined dichotomization of rural versus urban spaces, which fails to capture the complex mosaic of infrastructural, social and environmental factors driving risk. Such evaluation is especially important for *Plasmodium* transmission and malaria disease. To improve targeting of anti-malarial interventions, a continuous composite measure of urbanicity using spatially-referenced data was developed to evaluate household-level malaria risk from a house-to-house survey of children in Malawi.

**Methods:**

Children from 7564 households from eight districts throughout Malawi were tested for presence of *Plasmodium* parasites through finger-prick blood sampling and slide microscopy. A survey questionnaire was administered and latitude and longitude coordinates were recorded for each household. Distances from households to features associated with high and low levels of development (health facilities, roads, rivers, lakes) and population density were used to produce a principal component analysis (PCA)-based composite measure for all centroid locations of a fine geo-spatial grid covering Malawi. Regression methods were used to test associations of the urbanicity measure against *Plasmodium* infection status and to predict parasitaemia risk for all locations in Malawi.

**Results:**

Infection probability declined with increasing urbanicity. The new urbanicity metric was more predictive than either a governmentally defined rural/urban dichotomous variable or a population density variable. One reason for this was that 23% of cells within politically defined rural areas exhibited lower risk, more like those normally associated with “urban” locations.

**Conclusions:**

In addition to increasing predictive power, the new continuous urbanicity metric provided a clearer mechanistic understanding than the dichotomous urban/rural designations. Such designations often ignore urban-like, low-risk pockets within traditionally rural areas, as were found in Malawi, along with rural-like, potentially high-risk environments within urban areas. This method of characterizing urbanicity can be applied to other infectious disease processes in rapidly urbanizing contexts.

**Supplementary Information:**

The online version contains supplementary material available at 10.1186/s12936-021-03950-5.

## Background

Considerable and diverse evidence demonstrates that health profiles differ between urban and rural areas across the globe, but especially in developing countries [1–14]. Underlying these differences are diverse, context-specific factors involving the social and built environment that characterize and differentiate urban and rural spaces, and thereby affect health profiles. Studies often use dichotomous classifications of urban and rural to explain differences in disease prevalence or incidence, but such designations have been usually created for governmental administrative purposes [15], and may be unrelated to topographical, infrastructural and economic patterns that might serve as important determinants of disease risk. Some studies have evaluated specific disease risk factors in relation to a derived urbanicity scale [16], while others have created multi-country urbanicity metrics based on composite geographic, economic and political variables [17]. Studies of health and urbanicity gradients have primarily considered chronic diseases [18–20], but recent research has started to examine infectious outcomes as well [21, 22]. As developing countries urbanize and potentially create new opportunities for infectious disease transmission and health [23], developing and testing new measures of urbanicity becomes critical.

The need for a multifactorial, continuous "urbanicity" metric that can be generalized across different settings has been recognized [22, 24], as it provides a more nuanced framework that captures the complexities of urban and rural environments and their impacts on health [25]. Vlahov and Galea [15] developed a theoretical framework for studying connections between urbanicity and health that included the social environment (spaces where people interact with one another through social and market exchanges), the physical environment (nature of the physical spaces where people live and work), and health and social services (access to medical care and interventions). Lourenco [26] argued for recognizing a mosaic of rural and urban spaces, and for the need to consider a rural-urban continuum. Although the framework of a rural-urban continuum is not widely used in disease pattern studies, the features that contribute to a more health-relevant definition of urbanicity have been noted. Cuba, for example, understood the need to provide health care services in isolated and remote areas that were comparable in quality to those found in more affluent urban centres [27]. This has resulted in improved health profiles in isolated areas. Similarly, rural areas in Kenya with access to telecommunications services (e.g. cell phones and mobile banking) have significantly better health profiles than other, nearby, equally rural areas that lack these services [28]. Indeed, features associated with urbanicity may have more complex, indirect relationships to health. The building of improved transportation infrastructure in rural areas, for example, might increase the economic profile of rural communities, but at the same time lead to increased road accidents [29–32]. Thus, a comprehensive, continuous measure of urbanicity should comprise relevant factors that differentiate rural and urban spaces in a manner that is associated with disease risks or health outcomes of interest. Some generic characteristics of such a metric are likely to be similar; however, details of what is relevant will vary with the ecology and epidemiology of each disease. Ultimately, such an urbanicity measure might help improve forecasting capacity and specificity to prevention or control.

Any robust measure of urbanicity would ideally allow for both within- and among-country comparisons. Studies with a scaled measure of urbanicity typically have relied on local survey data to create this construct [33, 34]. While comprehensive, these studies lack generalizability and thus require local resources to collect the necessary data. A metric that uses readily available, spatially-referenced, Geographic Information System (GIS)-based data layers (e.g. census, environmental, economic) could be generalizable, while providing increased specificity for targeted interventions to high-risk regions.

Malaria is an example of a disease whose risks differ between rural and urban settings, and whose control strategies would benefit from a more nuanced understanding of which “rural” features might actually be protective, and which “urban” features could represent risk. Incidence of malaria is generally lower in urban compared to rural areas [25, 35, 36]. Differences in malaria incidence between urban and rural spaces are partly explained by fewer opportunities for mosquito vector reproduction in urban areas and reduced blood feeding on humans [37–39]. Urban habitats are considered generally less favourable for most competent *Anopheles* species due to the paucity of suitable breeding sites [40, 41]. In addition, urban house structures tend to restrict access of adult mosquitoes to humans at night, thus reducing transmission [42]. However, despite historical reports indicating that malaria is a “rural” problem, this disease is considered to be an emerging threat in rapidly urbanizing areas of sub-Saharan Africa [43, 44].

These complex relationships between malaria risk and urbanicity suggest that, in addition to malaria control programs that have promoted insecticide treated nets (ITNs), improved diagnostics and vector control, recent reductions in malaria incidence and mortality might be partly attributable to rapid urbanization and development [45]. The lines between what were formerly considered rural and urban have become increasingly blurred; stronger connections between the city and country through human movement, increased “citylike” infrastructure in rural areas, and increasing population density in peri-urban areas have rendered a dichotomous classification obsolete [21]. Factors that characterize very large urban settings are found within smaller urban and partly rural settings (e.g., improved housing, accessible and available health care, greater population density, as well as access to transportation and markets). Conversely, characteristics associated with rural settings, such as small-scale cropping, standing water, poor housing, and inadequate health services, can also be found in some urban areas. A more thorough understanding of how specific features of urbanization could influence *Plasmodium* transmission across the urban-to-rural landscape gradient should improve the effectiveness and efficiency of targeted interventions.

To develop and apply a new urbanicity measure relevant to countrywide malaria risk in Malawi, the Lourenco [26] and the Vlahov/Galea [15] frameworks were integrated by creating a continuous, composite, scaled measure of urbanicity that comprises aspects of the social environment, the physical environment, as well as health and social services access. Using this composite urbanicity measure, the manner in which rural-like environments exist within, and proximate to, urban areas was analysed, as well as how urban-like contexts have emerged within otherwise rural settings. Malaria, a disease known to vary between urban and rural contexts, was evaluated for whether there are consistent and predictable patterns of risk along an urbanicity gradient.

## Methods

### Household survey and child infection data

The survey involved 7564 households in eight Districts throughout Malawi, conducted during April/May 2007 [46]. Within each District, 16 to 30 Enumeration Areas (EAs) were chosen at random. Within each EA, 10 to 81 households were randomly selected. The geographic location of each sampled house was recorded using a GPS unit. After obtaining informed consent from the head of household, demographic information on all household members was collected using a standard questionnaire. In addition, one child between 6 and 59 months of age was identified for inclusion in the survey. Although household and individual malaria risk data were gathered from all households, only 4684 households where eligible children were present were selected for testing for *Plasmodium* infection. In households with more than one eligible child, only one was selected for malaria testing. A finger-prick blood sample was taken and later examined by a trained microscopist for presence of any species of *Plasmodium*. There was no attempt to identify the *Plasmodium* species, but a majority of infections in Malawi are known to be *P. falciparum* [47]. See Fig. 2 for locations of included households and parasitaemia status of selected children.

Information on household-level material assets collected by project enumerators included: type of house construction, water and light sources, type of toilet facilities, and presence of livestock, electronic goods and vehicles (bicycles, motorcycles and cars). All information was entered into a Microsoft Access database. Principal Components Analysis (PCA) was applied to these household-level material items. PCA [48] is a data dimension reduction technique that, given a set of p, possibly correlated, variables yields p new, uncorrelated, and mutually orthogonal variables, called Principal Components (PCs). Each component is defined as a linear combination of the original p variables, with the coefficients of the original p variables in the linear combination called factor loadings. The PCs are ordered so that the first PC accounts for the largest amount of variation in the data, and the subsequent components explain a lesser amount. Dimension reduction via PCA is achieved by replacing the original p variables with an appropriate number r, where r ≤ p, of PCs that explain a significant percentage of the total variation in the data. The first PC was selected as a composite measure of material wealth and socio-economic status (SES) and divided into quintiles. The SES quintiles, representing SES "classes" for each household were then added to the database [49].

### Data sources and urbanicity measure creation

To create a measure of urbanicity that does not require detailed, on-the-ground surveys, easily obtained, publicly available data were analysed, based on sources that could allow for cross-country comparisons. Six environmental, social, or infrastructural features commonly considered relevant to malaria risk, and that often differentiate urban and rural areas, were considered: population density, transportation infrastructure, and location of health services along with elevation and proximity to surface water (rivers and lakes). These components relate to the social environment, to the social/health services associated with the urban-to-rural continuum of interest and to environmental factors that characterize rural regions in this context [15]. Increased population, proximity to roads and health services all characterize development, access to areas of economic activity and the ability to receive government services. Low elevation and proximity to water characterize extremely rural and undeveloped regions in this context such as areas along Lake Malawi or in the low lying regions along the border with Zambia [50].

**Fig. 1:**
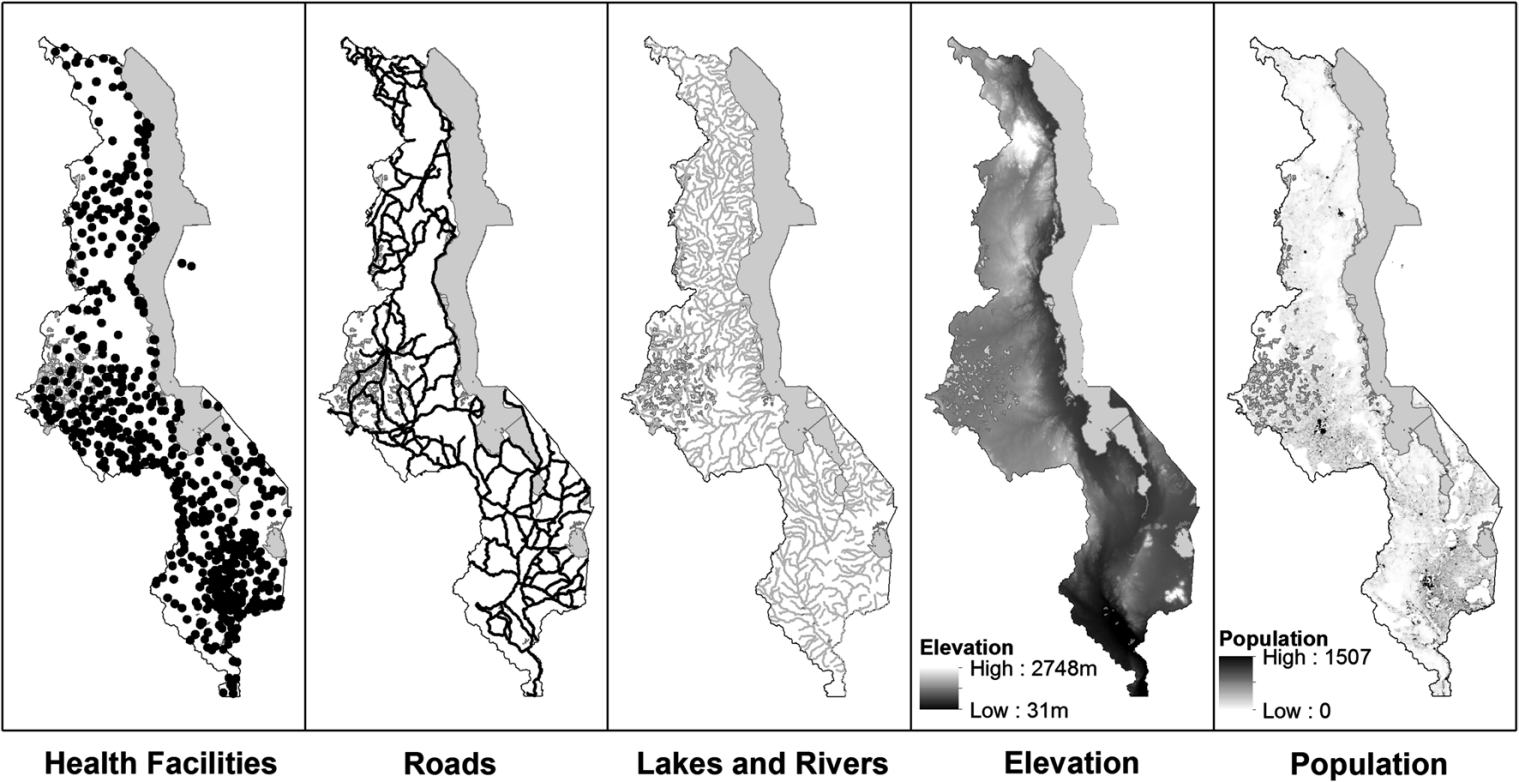
GIS layers for locations of health facilities, roads, water (rivers and lakes), elevation and population

Spatially-referenced data for elevation, water bodies, and transportation networks in Malawi were downloaded from the website of DIVA-GIS [51]. Mean population within a 1 km buffer around each household location was extracted from a 30 m resolution raster-based composite of census and remotely sensed data on locations of human settlements obtained from the WorldPop Project [52]. These environmental and population data were integrated into a GIS database using ArcGIS [53] (Fig. 1). In addition, both public and private health facility locations in Malawi, that had been comprehensively surveyed in 2003 by the Japan International Cooperation Agency (JICA), were obtained. The geo-coordinates for each health facility, as well as the health facility type, ownership and funding source, were included in the database [54].

For point features (e.g. health facilities), point-to-point distances were calculated. For linear features (e.g. roads), distance to the nearest point on the closest line was calculated. Computing distances using the Euclidean distance formula provides measures that some research has shown to be reasonable proxies for distances generated by other, more complex methods that measure actual travel routes [55, 56]. However, other studies have suggested that measures of access to health services based on Euclidean distances can overestimate access for a significant percentage of a given population compared with more complex metrics (e.g. road networks) due to underestimation of true travel distances [57]. Though Euclidean distances tend to underestimate true travel distances, they are correlated with true travel distances [58, 59]. Given this finding, and lacking information on actual travel routes from households to health facilities, Euclidean distances were used as a proxy for travel times required to receive health services.

From the human population data layer, 1 km buffers around each point of interest were created, and the mean population within each buffer was extracted and recorded. To construct a measure of urbanicity for the entire country of Malawi, a 1 km grid of points covering Malawi was constructed. Distances from a household, or any other point of interest, to any other point, line, or polygon feature were calculated, and raster values were extracted based on GPS location.

To derive the composite measure of urbanicity, PCA was applied to the six gridded variables that were considered relevant to the multi-dimensional concept of urbanicity, namely, distance to health facility, road, lake and river, as well as population and elevation, all appropriately normalized. Each household was assigned the values of the PCs relative to the grid centroid closest to its location.

### Statistical analyses

To investigate specific patterns of association between features of urbanicity and *Plasmodium* infection, controlling for potential confounders, multivariate logistic regression models were developed. To assess whether the proposed composite measure of urbanicity provided a better explanation of *Plasmodium* infection than other readily available indicators of urbanicity, such as population density or a binary indicator of urban *vs.* rural areas obtained from government statistics, three logistic regression models were fitted. The three models regressed the logit of observed malaria occurrence on: (i) the composite urbanicity measure defined by the first and second Principal Components; (ii) population density; and (iii) a binary urban/rural indicator. The three models were compared based on their Akaike’s Information Criteria (AIC) value [60], recognizing that the model with the lowest AIC is the model that best fits the data and better explains the variability in the observed occurrence of infection. In addition, receiver operating characteristic (ROC) curves [61] were developed to compare model performance between the continuous measure produced in this research and the dichotomous measure. All analyses were performed using R (ver. 3.5.1) statistical software [62].

## Results

### *Plasmodium* infection survey

**Fig. 2:**
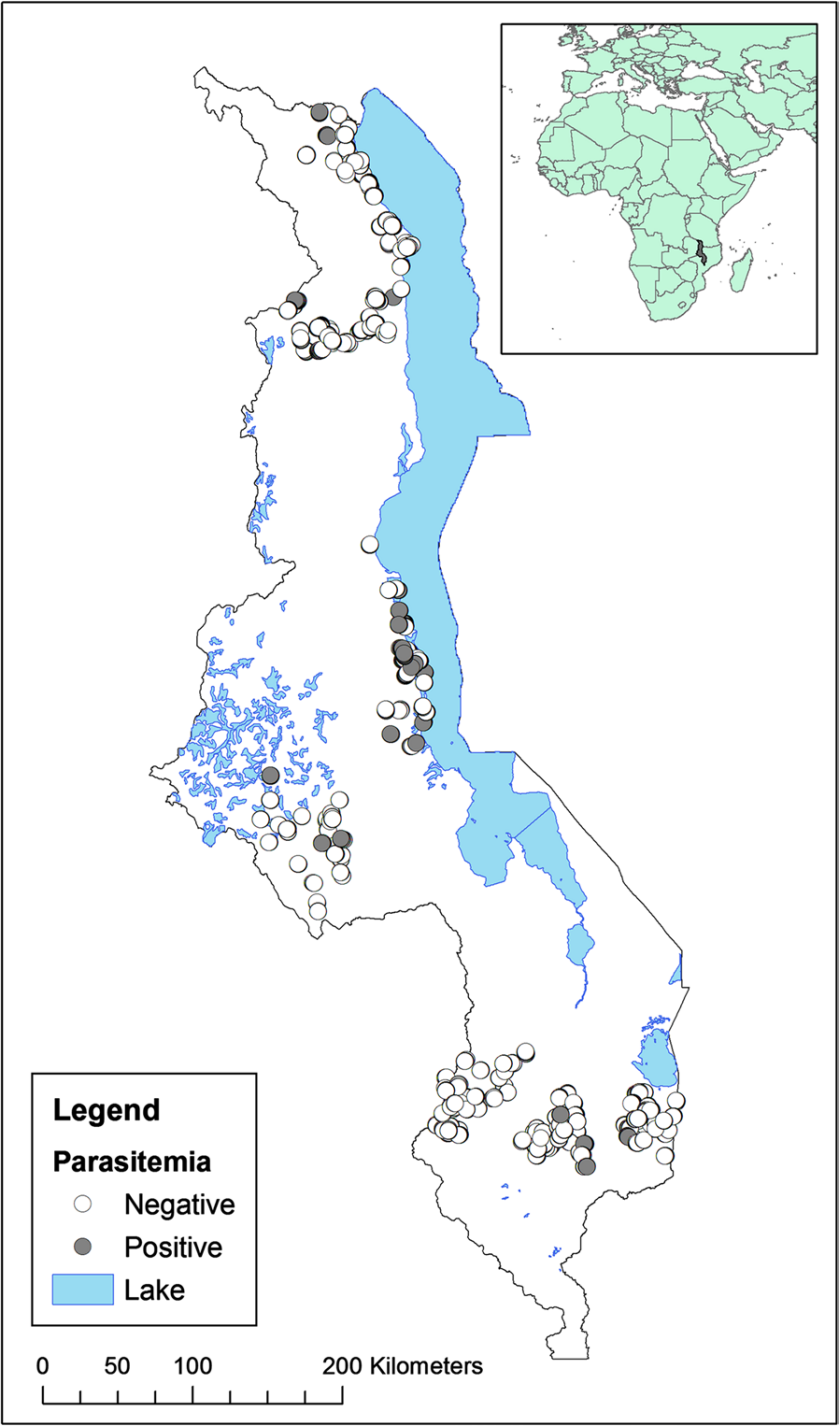
Maps of Malawi showing locations of households and the parasitemia status of the child in each household in the Northern, Central and Southern Regions surveyed during the 2007 sampling period

Of 4684 children tested for *Plasmodium* infection, 966 (20.6%) were found to be slide-positive (Fig. 2). More than half (57%) of these children reported sleeping under an ITN the previous night (Table 1). The mean age of children tested for infection was 17.1 months (Table 1). Children who were tested were younger and slightly poorer compared to those who were not tested (Additional file 1: Table S1).

**Table 1:** Characteristics of children and their households, aggregated and stratified by *Plasmodium* infection in eight Districts of Malawi, 2007

	Total Sampled	*Plasmodium* Negative	*Plasmodium* Positive	
	N = 4684	N = 3718	N = 966	OR [95% CI]
Slept under ITN (fraction)	0.57 (0.50)	0.60 (0.49)	0.47 (0.50)	0.59 [0.52;0.69]
Age (months)	17.1 (6.86)	16.9 (6.83)	18.0 (6.90)	1.02 [1.01;1.04]
Male (fraction)	0.50 (0.50)	0.50 (0.50)	0.52 (0.50)	1.09 [0.94;1.25]
Wealth quintile				
Ultra poor	1142 (24.4%)	810 (21.8%)	332 (34.4%)	Ref
Very poor	1104 (23.6%)	858 (23.1%)	246 (25.5%)	0.70 [0.58;0.85]
Poor	954 (20.4%)	776 (20.9%)	178 (18.4%)	0.56 [0.45;0.69]
Less poor	828 (17.7%)	719 (19.3%)	109 (11.3%)	0.37 [0.29;0.47]
Least poor	656 (14.0%)	555 (14.9%)	101 (10.5%)	0.44 [0.35;0.57]
Water source				
Borehole	2677 (57.2%)	2088 (56.2%)	589 (61.0%)	Ref
Piped into Yard	596 (12.7%)	504 (13.6%)	92 (9.52%)	0.65 [0.51;0.82]
Public Faucet	527 (11.3%)	484 (13.0%)	43 (4.45%)	0.32 [0.23;0.43]
Traditional public well	388 (8.28%)	245 (6.59%)	143 (14.8%)	2.07 [1.65;2.59]
Piped	281 (6.00%)	224 (6.02%)	57 (5.90%)	0.90 [0.66;1.22]
River/Lake/Canal	215 (4.59%)	173 (4.65%)	42 (4.35%)	0.86 [0.60;1.21]
Toilet type				
Pit latrine	3591 (76.7%)	2951 (79.4%)	640 (66.3%)	Ref
Other toilet	928 (19.8%)	667 (17.9%)	261 (27.0%)	1.80 [1.53;2.13]
Flush toilet	117 (2.50%)	72 (1.94%)	45 (4.66%)	2.88 [1.95;4.21]
Bush toilet	32 (0.68%)	15 (0.40%)	17 (1.76%)	5.22 [2.57;10.7]
Ventilated improved pit latrine (VIP)	16 (0.34%)	13 (0.35%)	3 (0.31%)	1.11 [0.24;3.49]
Roof material				
Grass	3386 (72.3%)	2588 (69.6%)	798 (82.6%)	Ref
Tin	1298 (27.7%)	1130 (30.4%)	168 (17.4%)	0.48 [0.40;0.58]
Floor material				
Dirt	3671 (78.4%)	2855 (76.8%)	816 (84.5%)	Ref
Cement	1013 (21.6%)	863 (23.2%)	150 (15.5%)	0.61 [0.50;0.73]
Elevation	788 (274)	800 (273)	706 (267)	1.00 [1.00,1.00]
Population	1.53 (4.31)	1.64 (4.59)	1.12 (3.01)	0.96 [0.94;0.99]
Distance to nearest				
Health facility	4.08 (3.16)	3.81 (2.90)	5.11 (3.84)	1.13 [1.11;1.15]
Road (km)	2.69 (3.41)	2.70 (3.53)	2.63 (2.91)	0.99 [0.97;1.02]
Lake (km)	24.7 (20.5)	27.7 (20.5)	13.1 (15.5)	0.96 [0.95;0.96]
River (km)	2.32 (2.09)	2.32 (2.11)	2.31 (2.03)	1.00 [0.96;1.03]
Urban or rural				
Rural	4249 (90.7%)	3346 (90.0%)	903 (93.5%)	Ref
Urban	435 (9.29%)	372 (10.0%)	63 (6.52%)	0.63 [0.47;0.82]

Univariate Odds Ratios (OR) compare parasitaemia positive versus negative children by each characteristic. All data are reported as counts unless specified

### Infection and geographic features

Children with *Plasmodium* infection lived further from the nearest health facility and closer to a lake body compared to those who tested negative (5.1 km vs. 3.8 km and 13.1 km vs. 27.7 km, p ≤ 0.001, respectively). Infected children, however, did not live closer to a road (2.6 km vs*.* 2.7 km, p = 0.6) nor to a river (2.3 km vs. 2.3 km, p = 0.9). Infected children resided in significantly less densely populated areas (z = 1.12 vs. z = 1.64, p ≤ 0.001), and at a lower elevation (z = -0.57 vs. z = − 0.28, p ≤ 0.001) than children who were not infected (Table 1).

### Urbanicity measure and government classification

**Fig. 3:**
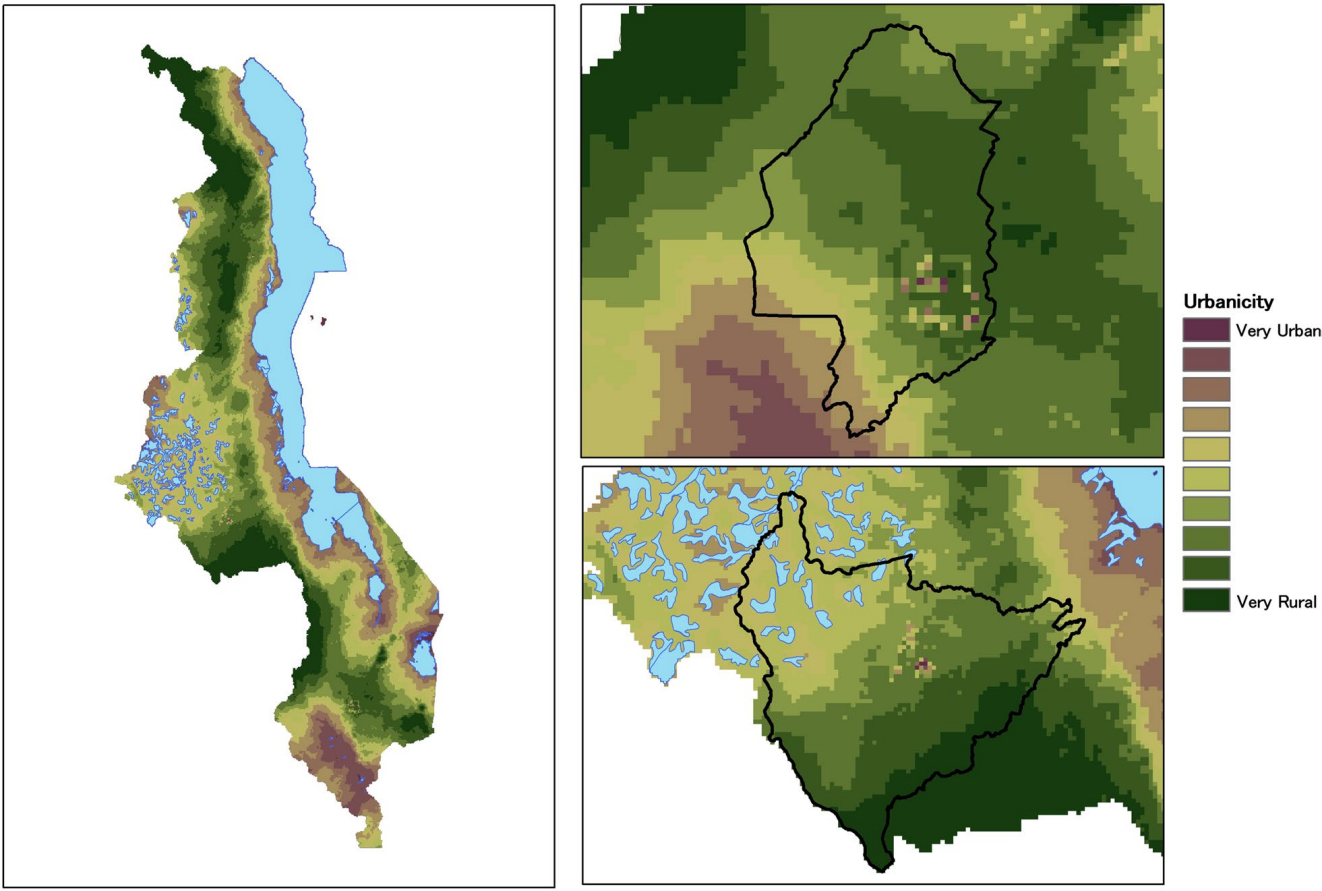
Urbanicity measure represented as the sum of the first two principle components for **A** all of Malawi, and in greater detail for the cities of **B** Blantyre and **C** Lilongwe. Higher values signify more urban

Results of the PCA showed that all six urbanicity variables were strongly represented in the first and second PCs (Table 2). In particular, the six variables tended to fall into two distinct groups. The first PC weighted more heavily those aspects of urbanicity that are related to human and infrastructural factors, such as population density, roads and health facilities. On the other hand, the second PC is a combination of variables involving biogeographic aspects of urbanicity, e.g. elevation, proximity to lakes, and proximity to rivers and streams, which should be important to vector mosquito abundance. The first two PCs together account for 49% of the total variation in the data and represent different aspects of urbanicity. Because these two PCs are both dominant and more interpretable than the remaining four, a composite measure of urbanicity was defined as the first two PCs. Figure 3 provides maps of how this urbanicity measure, representing the sum of the first two principal components, is geographically distributed throughout Malawi.

**Table 2:** Principal Component Analysis (PCA) loadings, percentage of variance explained, and cumulative proportion of variance explained by the six principal components

Variable	PC1	PC2	PC3	PC4	PC5	PC6
Distance to health facility	− 0.65	− 0.03	− 0.05	− 0.20	− 0.17	− 0.71
Distance to road	− 0.60	− 0.09	0.19	− 0.42	0.08	0.64
Distance to river	− 0.10	− 0.41	0.78	0.44	0.11	− 0.10
Distance to lake	0.04	0.66	0.35	− 0.20	0.61	− 0.18
Population	0.41	− 0.05	0.44	− 0.64	− 0.46	− 0.11
Elevation	− 0.18	0.63	0.20	0.37	− 0.61	0.17
Standard deviation	1.30	1.11	1.00	0.92	0.89	0.68
Proportion of variance	28.00%	20.50%	16.50%	14.10%	13.30%	7.70%
Cumulative proportion	28.00%	48.50%	65.00%	79.00%	92.30%	100.00%

The first two principal components are used in subsequent analyses as the urbanicity metric

This PCA-derived composite urbanicity measure was compared with official government urban and rural designations to assess whether the new measure identified “urban-like” spaces in areas designated as “rural” by the official classification, and conversely, whether there were “rural-like” spaces in areas officially designated as “urban.” Each household location was re-classified as “rural” if the urbanicity measure was below the median, or as "urban" if above the median. This new, PCA-derived, urban–rural re-classification was then compared with the official governmental designation of urban or rural to evaluate which household locations were classified differently. Nearly half (45.6%) of the household locations defined as rural by the Malawian government were classified as urban by the PCA-derived urbanicity measure. Likewise, there was heterogeneity within the survey areas that were officially defined as urban, with 4.4% of study households (all within the two largest cities) being classified as rural according to the urbanicity-measure driven classification.

### Relationships of urbanicity to *Plasmodium* infection

**Fig. 4:**
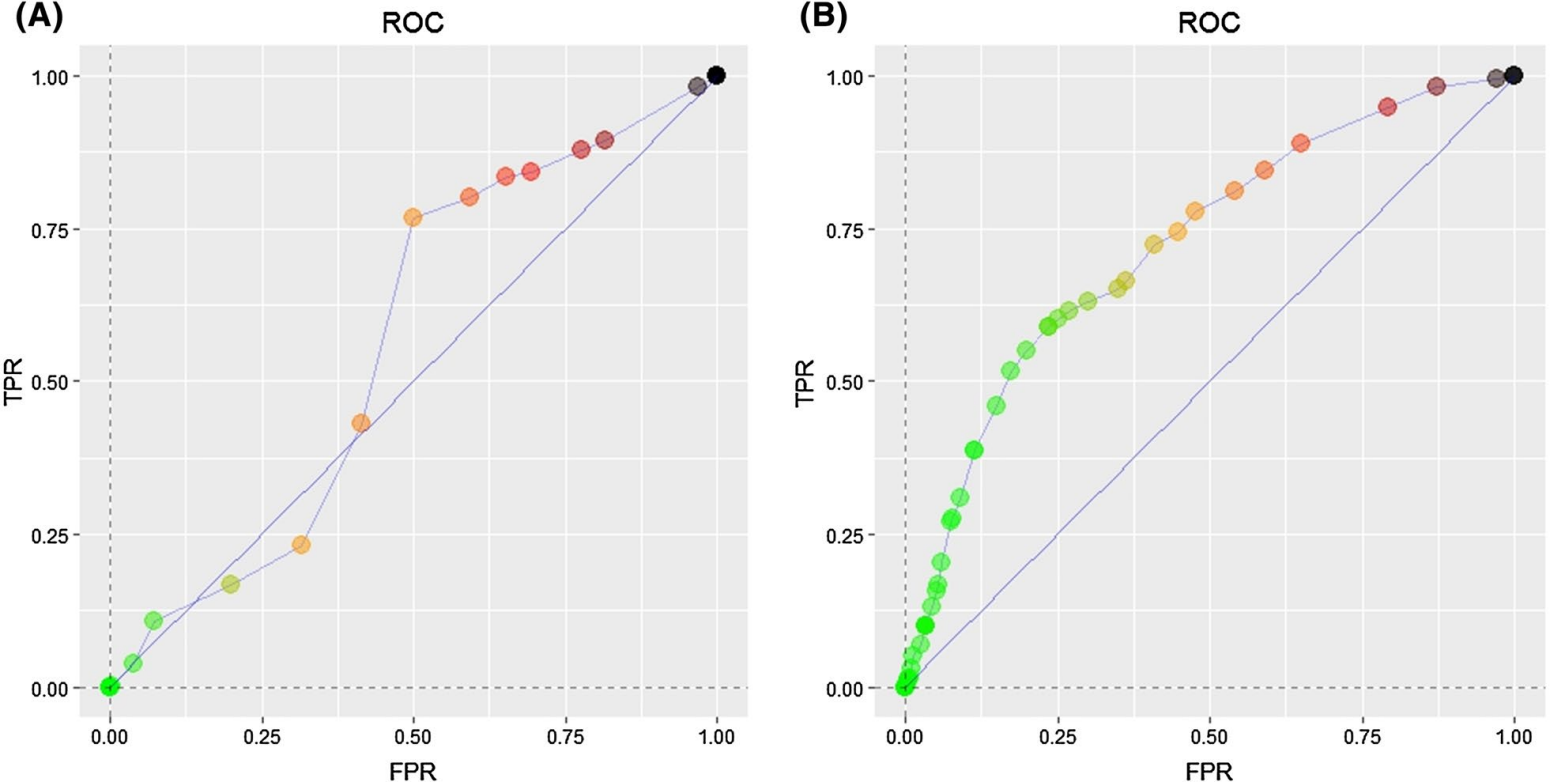
ROC curves comparing predicted parasitemia for households classified using **A** the dichotomous measure of urban and rural from the government-sponsored census and **B** the PCA-weighted composite measure

To assess the predictive utility of the PCA-derived composite measure, it was compared with the dichotomous urban–rural classification in the Malawi census data using receiver operating characteristic (ROC) curves (Fig. 4). For the government dichotomous classification, the curve mostly rests on the diagonal, while for the PCA-derived composite, the curve extends closer to the upper left corner. This indicates that the PCA composite classification is a superior predictor of *Plasmodium* infection compared to the official census-based measure.

### Regression-based associations

Logistic regression models indicated that ITN use was highly predictive of reduced infection risk, and there was no difference by gender (Table 3). Using the poorest SES quintile as a reference, SES was inversely related to *Plasmodium* parasitaemia, with the wealthier groups experiencing the lowest likelihood of testing positive. In other univariate logistic regression models, age, distance to nearest health facility, distance to lake, and population density were all significantly associated with parasitaemia, but distance to river and road were not (Table 3).

**Table 3:** Univariate and multivariate models of parasitaemia risk

	Model 1 with standard predictors			Model 2 after backwards selection			Model 3 with urbanicity measure		
Predictors	Odds Ratio	CI	p	Odds Ratio	CI	p	Odds Ratio	CI	p
(Intercept)	0.23	0.15–0.34	< 0.001	0.29	0.22–0.38	< 0.001	0.53	0.38–0.73	< 0.001
Slept under ITN	0.43	0.36–0.52	< 0.001	0.63	0.55–0.73	< 0.001	0.44	0.37–0.54	< 0.001
Age	1.03	1.01–1.04	< 0.001	1.03	1.02–1.04	< 0.001	1.03	1.02–1.05	< 0.001
Gender	1.08	0.91–1.29	0.388	1.02	0.89–1.19	0.74	1.1	0.92–1.32	0.287
Water Source									
Piped into Yard	0.9	0.61–1.32	0.581				1.09	0.72–1.63	0.69
Public Faucet	0.22	0.13–0.37	< 0.001				0.24	0.14–0.40	< 0.001
Traditional Public Well	1.73	1.30–2.31	< 0.001				1.01	0.76–1.36	0.932
Piped	1.2	0.75–1.94	0.447				1.1	0.67–1.78	0.71
River/Lake/Canal	0.73	0.49–1.11	0.139				0.82	0.54–1.24	0.354
Tin Roof	0.64	0.42–0.97	0.035				0.6	0.40–0.91	0.016
Cement floor	1.83	1.13–2.99	0.015				1.72	1.06–2.80	0.029
Wealth									
Very poor	0.83	0.66–1.05	0.13	0.76	0.63–0.92	0.006	0.89	0.70–1.13	0.344
Poor	0.75	0.56–1.00	0.048	0.61	0.49–0.75	< 0.001	0.76	0.57–1.03	0.073
Less poor	0.37	0.23–0.59	< 0.001	0.39	0.31–0.50	< 0.001	0.44	0.27–0.71	0.001
Least poor	0.51	0.25–1.05	0.068	0.47	0.37–0.60	< 0.001	0.57	0.27–1.21	0.141
Distance to nearest									
Health Facility	1.04	1.01–1.07	0.004	1.02	1.00–1.04	0.079			
Road	0.98	0.95–1.02	0.271	1.01	0.98–1.03	0.549			
Lake	1.01	1.00–1.01	0.004	0.99	0.99–0.99	< 0.001			
River	1.19	1.13–1.26	< 0.001	1.09	1.05–1.13	< 0.001			
Elevation	1	1.00–1.00	0.007						
Population	1	1.00–1.00	0.014						
PC 1 PC 2							0.97	0.97–0.98	< 0.001
Observations	2811			4684			2811		
Cox & Snell’s R2 /									
Nagelkerke’s R2	0.130 / 0.186			0.045 / 0.070			0.169 / 0.243		

Multivariate model 1 includes the individual components of the composite urbanicity measure except for the last two variables. Here, component 1 and 2 were included in one model. Multivariate model 2 includes the continuous urbanicity composite measure

To evaluate these relationships further, all covariates were included in a single multivariate logistic regression model (Table 3). Again, gender and distance to river were not significantly associated with parasitaemia risk when controlling for all the other covariates. ITN use was still protective against infection, although at a reduced magnitude. After accounting for other spatial indicators, the association between SES and infection disappeared. Older age, increased distance to health facilities and roads, decreased distance to lake, and increased population density were all associated with an elevated risk. A model including only the composite urban–rural measure indicated that increased levels of urbanicity were associated with decreased risks of being positive for *Plasmodium* infection (Table 3). Based on AIC values for logistic regression models with binary outcomes of parasitaemia status, the composite urbanicity measure performed better (AIC 4347) than one with a single predictor for population density (AIC 4758) and also better than one with only the urban/rural indicator variable (AIC 4760.)

Malaria risks in Malawi are heterogeneous within politically defined urban and rural areas. For example, 23% of rural survey locations exhibited low risk characteristics of an urban designation (based on the lower 25th percentile probability of parasitaemia). On the other hand, 4% of the urban survey locations exhibited high risk characteristics of a rural designation (based on the upper 25th percentile of the probability of parasitaemia.).

### Predicted malaria risk based on the composite measure

**Fig. 5:**
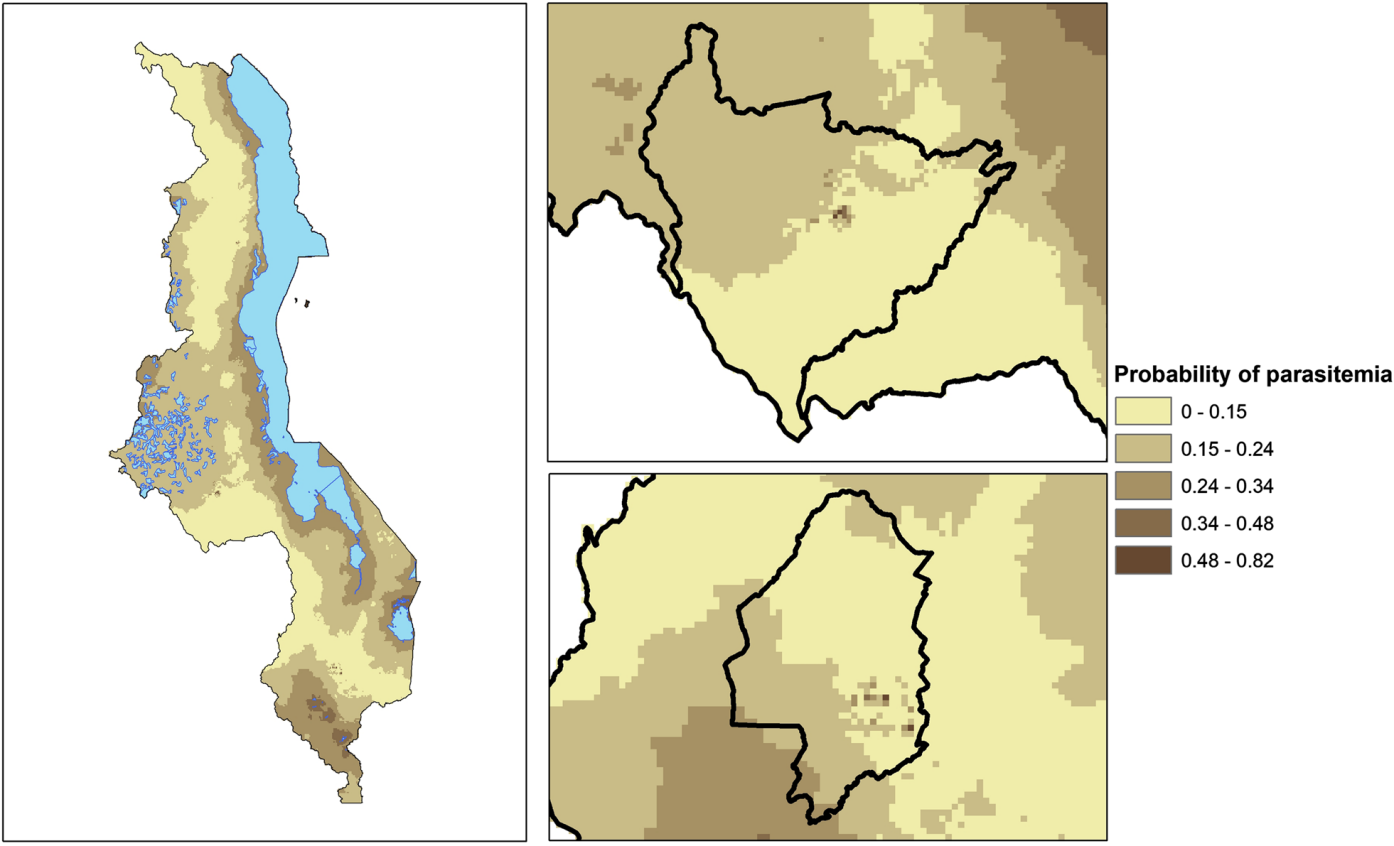
Spatial distribution of predicted *Plasmodium* infection risk given the PCA-derived composite measure of urbanicity

**Fig. 6:**
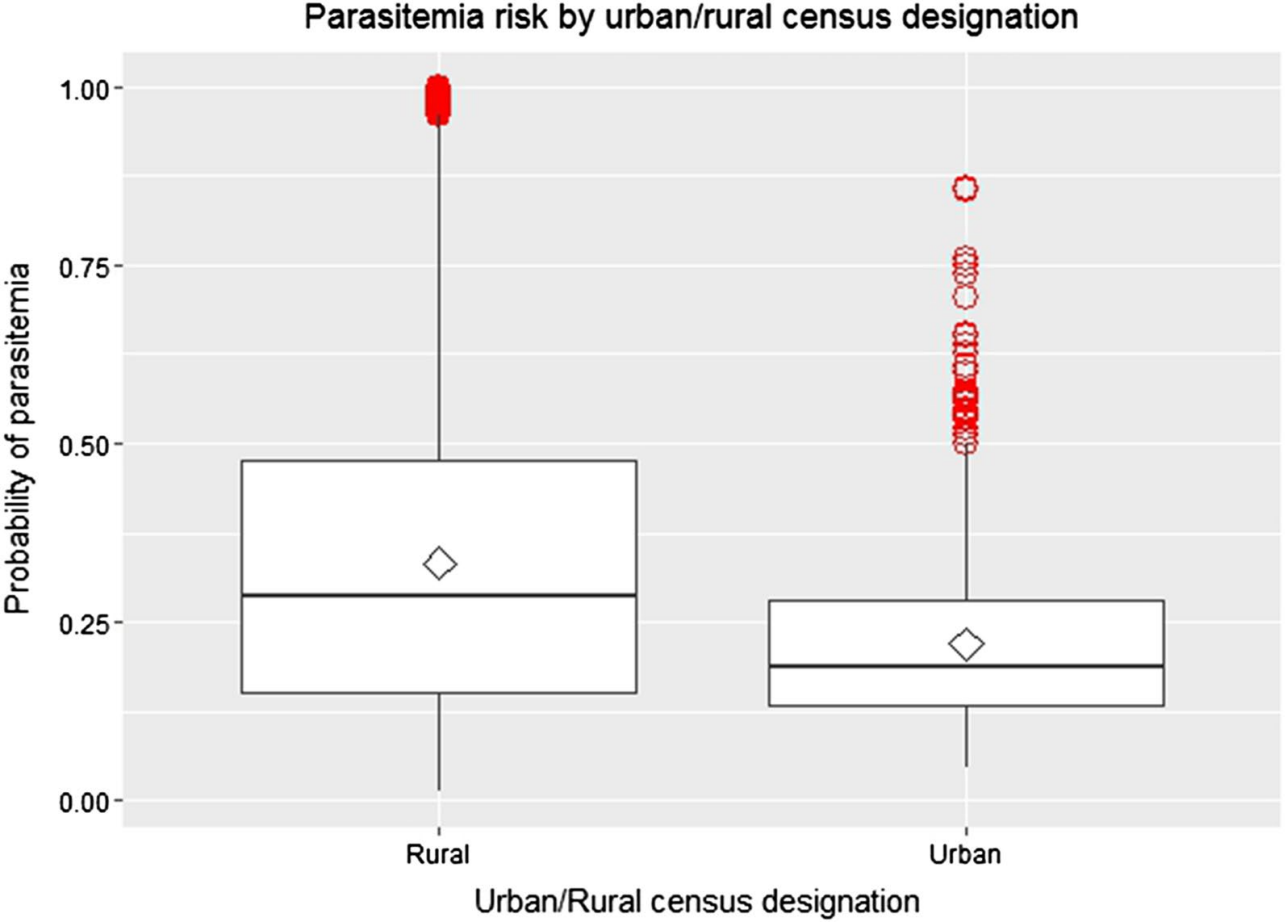
PCA-derived composite *Plasmodium* infection risk boxplots for households designated as urban or rural by official government classification

Using the composite measure of urbanicity and logistic regression, infection risk was calculated at each grid point over the entire map of Malawi (Fig. 5). Although areas along the lake were “high risk,” low lying areas of low population density such as those in and around the wildlife parks also had elevated levels of malaria risk. However, the prediction errors in these areas, where data are lacking, were very high. Finally, the estimated infection risk for households based on the PCA-derived composite measure was compared with the official census-based urban/rural designation of household location (Fig. 6). While the mean composite measure risk for rural areas was higher than that for urban areas, there was considerable overlap between the two, particularly at the second quartile. Many outlier household location points designated as “urban” had risk equivalent to points at the high end of “rural” risk. Indeed, 45 household locations designated as “urban” had *Plasmodium* infection risk equivalent to that of the highest quantile of risk among “rural” areas. Likewise, 295 households designated as “rural” had infection risk equivalent to that of the lowest risk among households designated as “urban.”

## Discussion

The analyses of this study applied different methods that created a more informative and relevant geosocial classification of malaria risk along an "urban-to-rural" continuum, indicate that there is a graded relationship with urbanicity. Indeed, the results suggest the value of creating a more complex, nuanced characterization of urbanicity when assessing *Plasmodium* transmission risk than what a classical binary classification offers. There are a number of special features in the results that are worth noting.

The analyses found that while *Plasmodium* parasitaemia status was strongly associated with distance to lakes, this was not so with proximity to rivers. Lakefronts may provide breeding opportunities for Anopheles, particularly *Anopheles funestus* [63, 64]. In addition, lake water is generally more seasonally stable in volume than that of rivers. However, the water layers used for this study did not include information on river width, depth or degree of water flow. A more comprehensive map of waterways for Malawi could yield more detailed results. Different river widths create environments that are more or less amenable to *Anopheles* species-specific reproduction, and might include conditions that are similar to lake and other still water bodies.

Other community-level factors such as greater distances to health services and roads were also found to be important risk factors (Table 3). Appreciating these community contexts, and thereby identifying specific areas at higher potential risk, will be important to developing more focused prevention. This process will lead to a better understanding of the causal mechanisms underlying risk and may unveil the manner in which closer proximity to certain features of more urbanized environments mitigates against *Plasmodium* infection in children.

In addition to the community-based urbanicity metric, various household-level factors, such as greater material wealth (a measure of SES) and the use of ITNs, were also found to be associated with reduced malaria risk in children. The analysis suggests that higher SES, greater ITN use, and living in more urbanized contexts each acted independently to decrease malaria risk. Thereby, the important household-level causal pathways appear to be more than house construction and condition, as is often posited. Considerably more work is needed to untangle the relationships between household- and community-level factors, and their relationships with malaria. The issue of urban–rural classification, a community-level construct, will become ever more salient as sub-Saharan African (SSA) countries continue to develop, and urbanization continues to expand.

This study and others have demonstrated the limitations of using a politically defined urban–rural construct. Within a politically defined urban or rural environment, there exists heterogeneity with respect to geographic, economic and socio-political risk factors. In general, the urban designations in use are not consistently defined across the countries of SSA. As this study in Malawi has shown, where fewer areas are classified as “urban”, people might be considered residents of rural areas even though their communities share indicators of urban settings. The reverse is also true. As other researchers have noted, independent of population density or remoteness, some “rural” communities experience better standards of living, increased economic opportunities, and city-like services such as health care and schools [15]. Inversely, small-area environmental conditions (family gardens, lowland springs, river edges) in urban settings might create highly suitable breeding sites for *Anopheles* vectors of malaria, much like what is typically thought of as rural.

The limitations of using a dichotomous definition of urbanicity have practical implications. When providing anti-malaria interventions, some “rural” communities may have areas with low disease risk, yet receive extensive prevention support, leading to a waste of resources that could be better targeted elsewhere. Similarly, rural-like areas near large and dense human settlements may be overlooked during anti-malaria interventions. Uneven geographic distribution of malaria risk and inadequate knowledge of the locations of transmission foci have been recognized as challenges to the spatial targeting of malaria control [54] in settings of heterogeneous transmission across geographic locations [65]. Results from the present study confirm that the transmission profile throughout a holoendemic country like Malawi varies considerably over space. These same analyses offer measurable and easily obtained markers that could potentially be used to aid in efficient targeting of anti-malaria interventions.

Another important aspect of the urban–rural continuum involves the level of connectivity between more rural and more developed contexts. In Malawi, however, ease of transport between areas along the developmental gradient is challenging. One study showed that people in extremely rural areas must rely on animal-drawn carts to receive basic medical services and must spend money to reach better secondary and tertiary care facilities located in larger cities and towns [66]. Transportation expenses have been shown to be a major barrier to receiving prompt care for serious health conditions in other African contexts [67]. Travel distance has been shown to be an obstacle to obtaining HIV care in Malawi [68]. While the present research used a simple measure of access to roads and health facilities as a proxy for spatial access to areas that provide economic opportunities and government services, future efforts might employ more detailed measures that take into account different types of transport infrastructure or travel times.

To further refine the use of a continuous urbanicity metric, at least three major areas need further consideration. First, although this study created a malaria-relevant urbanicity measure based on data that are readily available for any developing country, the lack of more specific markers of social and ecological contexts and economic development hampered the analysis. Data on the locations of schools or markets, for example, might have helped to make the results more focally accurate. Given the experiences and knowledge of the authors in Malawi, however, this analysis suggests that the composite measure is a reasonable representation of the gradient of urbanicity in Malawi. Second, associations with river and lake locations may not to be generalizable for cross-country comparisons. With some exceptions, colonial powers tended to establish SSA cities far from swamps and fresh water bodies. Residents of rural areas, on the other hand, often live near surface water. Even within cities, wealthier areas tend to be located at higher elevations and away from areas where water usually collects. These SSA generalities stand in contrast to European contexts which tended to favor locations amenable to trade by water routes. Third, the cross-sectional malaria data that were used may ignore important temporal effects of seasonal malaria. Seasonal transmission of *Plasmodium* has been demonstrated in Malawi in other studies [69, 70], although transmission does occur throughout the year. Data used in the present study were collected to assess infection prevalence toward the end of peak transmission, but seasonal patterns of transmission may also differ by urbanicity and developmental context. Regardless, seasonal variation cannot confound the relationship between urbanicity and parasitaemia, because the urbanicity measure does not vary in time in this analysis. It is recognized, however, that the data were collected in a single year and may not be representative of other years.

## Conclusions

Policy makers need more accurate classification of urban and rural spaces to make better use of limited intervention resources [24]. Control and prevention strategies that inappropriately target all “rural” areas may unnecessarily expend costly resources where they are not needed, while underserving truly remote and disconnected areas that face crushing malaria incidence and mortality [36, 43, 71–77]. The complement to this involves “urban” areas that are ignored because of a governmental designation. Malaria control policies are likely to be more cost-effective if governments encouraged a more scientific, context and disease-specific analysis of the complex urban–rural continuum. The analysis of measures associated with urbanicity in this Malawi study demonstrated a rural–urban gradient of associated malaria risk, but with urban-like pockets in areas traditionally classified as rural, and vice versa. Additionally, malaria risk showed a graded association with levels of the new urbanicity metric that were developed. Governments might encourage use of such an approach in developing policies to more effectively target anti-malaria interventions to populations with scarce resources.

## Supplementary Information

**Additional file 1: Table S1.** Univariate and multivariate models of parasitaemia risk. Multivariate model 1 includes the individual components of the composite urbanicity measure except for the last two variables. Here, component 1 and 2 were included in one model. Multivariate model 2 includes the continuous urbanicity composite measure

## Data Availability

The datasets used and/or analysed during the current study are available from the corresponding author on reasonable request. All code used for analysis is available in the kambanane/Urbanicity repository (URL: https://github.com/kambanane/Urbanicity).

## Abbreviations

AIC: Akaike’s Information Criterion
EA: Enumeration Area
GIS: Geographic Information System
ITN: Insecticide treated net
PC: Principal Component
PCA: Principal Components Analysis
ROC: Receiver Operating Characteristics
SES: Socio-economic status
SSA: Sub-Saharan Africa
