# Supplementary material for: An urban-to-rural continuum of malaria risk: new analytic approaches characterize patterns in Malawi
Source: Malar J. 2021 Oct 24;20:418. doi: 10.1186/s12936-021-03950-5 (PMC8543962; doi:10.1186/s12936-021-03950-5)
Supplement: Supplementary file 1 — Additional file 1: Table S1. Univariate and multivariate models of parasitaemia risk. Multivariate model 1 includes the individual components of the composite urbanicity measure except for the last two variables. Here, component 1 and 2 were included in one model. Multivariate model 2 includes the continuous urbanicity composite measure [file 12936_2021_3950_MOESM1_ESM.docx]

**Table S1** Univariate and multivariate models of parasitaemia risk. Multivariate model 1 includes the individual components of the composite urbanicity measure except for the last two variables. Here, component 1 and 2 were included in one model. Multivariate model 2 includes the continuous urbanicity composite measure.

|  | Tested | Not tested |  | |
| --- | --- | --- | --- | --- |
|  | N=4684 | N=2880 | OR | p.ratio |
| Slept under ITN (fraction) | 0.57 (0.50) | 0.57 (0.50) | 1.00 [0.91;1.09] | 0.939 |
| Age (months) | 17.1 (6.86) | 38.1 (12.1) | 1.23 [1.22;1.24] | 0.000 |
| Male (fraction) | 0.50 (0.50) | 0.52 (0.50) | 1.06 [0.97;1.16] | 0.213 |
| Wealth quintile |  |  |  |  |
| Ultra Poor | 1142 (24.4%) | 616 (21.4%) | Ref. | Ref. |
| Very Poor | 1104 (23.6%) | 557 (19.3%) | 0.94 [0.81;1.08] | 0.354 |
| Poor | 954 (20.4%) | 559 (19.4%) | 1.09 [0.94;1.25] | 0.257 |
| Less Poor | 828 (17.7%) | 563 (19.5%) | 1.26 [1.09;1.46] | 0.002 |
| Least Poor | 656 (14.0%) | 585 (20.3%) | 1.65 [1.43;1.92] | *<*0.001 |
| Water source |  |  |  |  |
| Borehole | 2677 (57.2%) | 1530 (53.1%) | Ref. | Ref. |
| Piped into Yard | 596 (12.7%) | 277 (9.62%) | 0.81 [0.70;0.95] | 0.009 |
| Public Faucet | 527 (11.3%) | 374 (13.0%) | 1.24 [1.07;1.44] | 0.004 |
| Traditional Public Well | 388 (8.28%) | 263 (9.13%) | 1.19 [1.00;1.40] | 0.048 |
| Piped | 281 (6.00%) | 307 (10.7%) | 1.91 [1.61;2.27] | *<*0.001 |
| River/Lake/Canal | 215 (4.59%) | 129 (4.48%) | 1.05 [0.84;1.32] | 0.673 |
| Toilet  Pit Latrine | 3591 (76.7%) | 2019 (70.1%) | Ref. | Ref. |
| Other Toilet | 928 (19.8%) | 576 (20.0%) | 1.10 [0.98;1.24] | 0.099 |
| Flush Toilet | 117 (2.50%) | 235 (8.16%) | 3.57 [2.85;4.50] | 0.000 |
| Bush Toilet | 32 (0.68%) | 26 (0.90%) | 1.45 [0.85;2.44] | 0.170 |
| VIP | 16 (0.34%) | 24 (0.83%) | 2.66 [1.41;5.13] | 0.002 |
| Roof  Grass | 3386 (72.3%) | 1851 (64.3%) | Ref. | Ref. |
| Tin | 1298 (27.7%) | 1029 (35.7%) | 1.45 [1.31;1.60] | *<*0.001 |
| Floor |  |  |  |  |
| Dirt | 3671 (78.4%) | 2027 (70.4%) | Ref. | Ref. |
| Cement | 1013 (21.6%) | 853 (29.6%) | 1.52 [1.37;1.70] | *<*0.001 |
| Elevation | 834 (371) | 848 (366) | 1.00 [1.00;1.00] | 0.226 |
| Population | 65.4 (126) | 52.4 (56.7) | 1.00 [1.00;1.00] | *<*0.001 |
| Distance to nearest |  |  |  |  |
| Health facility | 6.45 (4.80) | 6.07 (4.43) | 0.98 [0.97;0.99] | *<*0.001 |
| Road (km) | 2.69 (3.41) | 3.17 (3.91) | 1.04 [1.02;1.05] | *<*0.001 |
| Lake (km) | 26.1 (21.9) | 27.4 (21.2) | 1.00 [1.00;1.00] | 0.010 |
| River (km) | 2.32 (2.09) | 2.18 (2.00) | 0.97 [0.94;0.99] | 0.004 |
| Urban or rural |  |  |  |  |
| Rural | 4249 (90.7%) | 2632 (91.4%) | Ref. | Ref. |
| Urban | 435 (9.29%) | 248 (8.61%) | 0.92 [0.78;1.08] | 0.320 |
